# A novel 3A-based indirect enzyme-linked immunosorbent assay for serological testing of Senecavirus A

**DOI:** 10.1128/spectrum.00779-25

**Published:** 2025-10-30

**Authors:** Xiang-hui Ling, Wu-ying Ruan, Hao-jie Ren, Biao Zhang, Jia-hao Cong, Wen-ju Yuan, Guan-shang Liu, Hui-hui Huang, Zhao-jiang Chen, Shi-chong Han, Wen-rui He, Gai-ping Zhang, Yu-hang Zhang, Bo Wan

**Affiliations:** 1 International Joint Research Center of National Animal Immunology, College of Veterinary Medicine, Henan Agricultural University 731518, Zhengzhou, Henan, China; 2 Animal Diseases Control and Prevention Centre of Kaifeng, Kaifeng, Henan, China; 3 Ministry of Education Key Laboratory for Animal Pathogens and Biosafety, Henan Agricultural University 70573, Zhengzhou, China; 4 Longhu Laboratory, Henan Agricultural University, Zhengzhou University 70573, Zhengzhou, China; 5 School of Advanced Agricultural Sciences, Peking University 538778, Beijing, China; Shandong First Medical University, Jinan, Shandong, China

**Keywords:** Senecavirus A, 3A protein, enzyme-linked immunosorbent assay, serological testing

## Abstract

Senecavirus A (SVA) can cause a contagious porcine disease characterized by vesicular lesions on the mouth and feet. Its clinical symptoms are extremely similar to those of porcine foot-and-mouth disease, swine vesicular disease, and vesicular stomatitis, making it difficult to distinguish in clinical diagnosis. SVA can infect pigs at all stages and cause acute death of newborn piglets, causing serious economic losses to the pig industry. In this study, a novel indirect enzyme-linked immunosorbent assay (ELISA) was established based on the SVA 3A nonstructural protein, which was equally sensitive to the immunofluorescence assay (IFA). In addition, the indirect ELISA showed no cross-reaction with positive sera of porcine reproductive and respiratory syndrome virus, classical swine fever virus, porcine circovirus type 2, pseudorabies virus, porcine astrovirus, African swine fever virus, and porcine epidemic diarrhea virus, exhibiting good specificity. The maximum serum sample dilution for detecting SVA-positive serum sample is 1:4,096. The coefficients of variation obtained from both intra-assay and inter-assay repeated experiments were less than 5%. Additionally, 173 porcine serum samples were tested using both the indirect ELISA method and commercial kits, with immunofluorescence analysis (IFA) employed as an auxiliary verification method to further confirm the accuracy of the test results. The results showed that the compliance rate of the indirect ELISA (100%) was higher than that of the commercially available ELISA kit (89.60%). The indirect ELISA established in this study showed more efficient and reliable performance for SVA serological testing, making it more suitable for long-term monitoring and prevention of the SVA epidemic.

**IMPORTANCE:**

SVA mainly causes vesicular lesions in pigs, bringing economic losses to the porcine industry. Currently, no commercial vaccine is available, so effective diagnostic methods are vital for the prevention and control of the disease. Serological testing, as a diagnostic method to detect specific antibodies against pathogens, can also be used to confirm viral infection in non-vaccinated animals. Previous studies have shown that in Senecavirus A (SVA)-infected pigs, the antibody level of the 3AB protein is significantly higher than the structural VP1 protein. Considering the molecular weight of the 3B protein was only 2.4 kDa, it is speculated that the 3A protein is a potential diagnostic marker for SVA serological testing. In this study, a novel 3A-based indirect enzyme-linked immunosorbent assay (ELISA) was established, showing a 100.0% coincident rate compared with immunofluorescence assay for clinical serum. Since most studies of vaccine development focus on structural proteins, the 3A-based ELISA also provides a potential tool for distinguishing between infected and vaccinated animals.

## INTRODUCTION

Senecavirus A (SVA), also known as Seneca Valley virus (SVV), is a non-enveloped, single-stranded, positive-sense RNA virus (1, 2). It belongs to the *Picornaviridae* family and is the sole member of the *Senecavirus* genus (1, 2). The virus can cause a contagious porcine disease characterized by vesicular lesions of the mouth and feet with symptoms similar to those of swine foot-and-mouth disease (FMD), swine vesicular disease (SVD), and vesicular stomatitis (VS) (1–4).

SVA was first discovered and isolated in 2002 from PER.C6 cell cultures in Maryland, USA (2). Since its discovery, it has been reported in multiple countries, including Canada, Brazil, USA, Colombia, Thailand, Vietnam, and China (5–14). It is primarily characterized by the appearance of vesicles on the mucous membranes of the mouth and nose, as well as on the hoof coronet (3, 5). Meanwhile, the incidence rate of this disease is relatively high, and pigs of all ages can be infected with this disease, especially newborn piglets aged 1–4 days, which often exhibit clinical symptoms such as weakness, anorexia, diarrhea, sudden death, etc., with a mortality rate as high as 70%–80% (3, 15–18). Adult pigs often exhibit subclinical symptoms such as increased body temperature, limping, lethargy, decreased feed intake, and vesicular lesions on the mouth, nose, and hooves, which greatly reduce the overall immune defense of the herd and pose challenges to disease prevention and control on the farm (1, 19).

SVA viral particles exhibit an icosahedral structure with a diameter ranging from 17 to 25 nm (20–22). The viral genome consists of a 6,543 nt open reading frame (ORF), as well as a 5´ untranslated region (UTR) and a short 3´ UTR with poly(A) tail (5, 20). This ORF is further segmented into four structural proteins (VP1-VP2-VP3-VP4) and seven nonstructural proteins (2A-2B-2C-3A-3B-3C-3D), conforming to the standard L-4-3-4 layout of *Picornaviridae* family genomes (5, 20, 21).

Since there is currently no commercial SVA vaccine available in China, SVA clinical testing plays a crucial role in preventing and controlling the spread of SVA in the country. Currently, the main diagnostic methods for SVA include the viral neutralization test (VNT), indirect immunofluorescence assay (IFA), polymerase chain reaction (PCR), loop-mediated isothermal amplification (LAMP), and enzyme-linked immunosorbent assay (ELISA) (22–26). VNT and IFA are often used as the gold standard for virus detection, but they are not only complex to operate, they also take a long time to perform (27–31). Although PCR and LAMP technologies have extremely high detection sensitivity, their application is still limited by some practical factors (32). PCR relies on an expensive thermal cycler, while LAMP, although it does not require a thermal cycler, still needs specialized constant-temperature equipment (32). Both methods usually require professional operation and have extremely strict requirements for the laboratory environment to avoid the risk of false-positive results due to aerosol contamination (24, 32, 33). These factors significantly increase the application costs of PCR and LAMP technologies. Compared to the aforementioned methods, the ELISA method boasts the advantages of straightforward and swift operation, high sensitivity, strong specificity, and high measurement throughput (5, 27, 34). However, there are obvious differences in ELISA detection methods for different viral proteins or antiviral protein antibodies, and problems such as cross-reaction are also faced (35, 36). Therefore, it is of great significance to establish a simple and efficient SVA ELISA antibody detection method using good target materials.

To date, most serological testing methods for SVA target antibodies against structural proteins, which may bring difficulties for distinguishing between infected and vaccinated animals (23, 27, 37, 38). Developing nonstructural protein-based serological testing may solve this problem (37). Many studies generally agree that VP1 is the most immunologically advantageous of all the proteins in the *Picornaviridae* family of viruses (5, 39). However, the kinetics of the presence and levels of SVA antibodies with SVA-inoculated porcine serum show that SVA-3AB protein has superior antigenicity compared with L, VP1, 2C, 3C, and 3D proteins (5). As one of the main nonstructural proteins of SVA, the 3A protein not only exists in its mature form, but also functions as a part of the precursor protein 3AB (40). Meanwhile, considering that the 3B protein (NAYDGPKKNSKPPGALSLMEMQ) has a small size of only 2.4 kDa, it is speculated that there is a better reactivity between the 3A protein and SVA-positive serum sample, and the detection effect established with anti-SVA 3A protein antibody as the detection target will be improved.

In this study, a novel indirect enzyme-linked immunosorbent assay was established based on the SVA 3A nonstructural protein. Compared with commercial kits, it was found that the indirect ELISA was more efficient in detecting SVA-positive serum samples. It provides a more efficient assay for serological monitoring of SVA.

## MATERIALS AND METHODS

### 3A protein, cell, and main reagents

SVA 3A nonstructural protein, Instituto Biologico-Rim Suino-2 (IBRS-2) cells, and SVA CH-HuB-2017 strain (GenBank accession no. QOJ79820.1) used in this study were stored in our laboratory (41). The protein, which was stored at a concentration of 1 mg/mL, was expressed using *Escherichia coli* BL21 (DE3) cells and purified by nickel-column affinity chromatography. The HRP goat anti-swine antibody was purchased from Proteintech (China). The FITC rabbit anti-swine antibody was purchased from Sigma. The IBRS-2 cells were cultured in Dulbecco’s modified Eagle medium (Solarbio, 11995, China) supplemented with 10% fetal bovine serum (Gibco, 10099, USA) at 37°C under a 5% CO_2_ atmosphere.

### Serum samples

African swine fever virus (ASFV) and foot-and-mouth disease virus (FMDV) positive serum samples were obtained from the China Veterinary Drug Inspection Institute (Beijing, China). The positive serum samples of porcine reproductive and respiratory syndrome virus (PRRSV), classical swine fever virus (CSFV), porcine circovirus type 2 (PCV2), pseudorabies virus (PRV), porcine astrovirus (PAstV), porcine epidemic diarrhea virus (PEDV), and SVA, as well as SVA-negative serum samples, were obtained during clinical serological testing. The 173 clinical serum samples tested in this study were obtained from a farm in Henan Province, China.

### Comparison of the indirect ELISA with the commercial kit

The double-antigen sandwich ELISA antibody detection commercial kit based on SVA structural protein (Lanzhou Shouyan Biotechnology Co., Ltd., Lanzhou, China) and established indirect ELISA method based on 3A protein were used to detect the same porcine clinical serum samples and, finally, the detection results were compared and analyzed.

### IFA

To verify the reactivity of clinical serum samples to SVA, IFA was performed in IBRS-2 cells infected with SVA. Approximately 12–24 h after infection, the cells were fixed with 4% paraformaldehyde for 30 min and subsequently incubated with 0.1% Triton X-100 to mediate cell permeabilization for 15 min. After blocking the cells with 5% bovine serum albumin (BSA) for 30 min, the porcine clinical serum samples, diluted 1:250 in PBS, were incubated with the cells at 37°C for 1 h. After washing five times with PBST, the cells were incubated with Anti-Pig IgG (whole molecule)-FITC antibody produced in rabbit (Merck, F1638, China) for another 1 h at 37°C. After staining with DAPI (Solarbio, C0065, China), imaging of the cells was performed using a fluorescence microscope (Olympus, Japan).

### Establishment of indirect ELISA method based on 3A protein

The optimal 3A protein coating concentration and the serum sample dilution were determined through checkerboard titration. The 3A protein was diluted (0.25–4 µg/mL) and coated onto 96-well microtiter plates. SVA-positive and SVA-negative serum samples at different dilutions (1:50–1:400) were incubated. Subsequently, we used carbonate buffer (0.05 mol /L, pH 9.6), phosphate-buffered solution (0.2 mol /L, pH 7.5), and Tris-hydrochloric acid buffer solution (0.1 mol /L, pH 7.5) as antigenic diluents, respectively, to determine the optimal antigenic diluents. The antigen is coated at 37°C for 3 h or 4°C for 20 h to determine the optimal coating conditions for the antigen. The optimal blocking solution was preliminarily determined from 1% and 5% skimmed milk, 1% and 5% bovine serum albumin (BSA), and 1% and 5% casein. To further determine the optimal concentration of the blocking solution, we tested 1%, 2%, 3%, 4%, and 5% of the optimal blocking solution at 37°C for 1 h, 37°C for 2 h, 37°C for 3 h, 37°C for 2 h, and 4°C for 20 h. The serum samples were incubated for 0.5, 1, 1.5, 2, and 2.5 h to select the optimal incubation time. The enzyme-labeled antibody was diluted at the ratios of 1:1,000, 1:2,000, 1:4,000, 1:8,000, 1:16,000, and 1:32,000, and then incubated for durations of 0.25, 0.5, 0.75, 1, and 1.5 h to determine the optimal reaction conditions. After four rounds of washing, the chromogenic solution was added and incubated for 5, 10, 15, 20, and 25 min at ambient temperature in the dark. Finally, 50 µL of 2 mol/L sulfuric acid solution was added to terminate the reaction, and the absorbance was measured at 450 nm using a microplate reader. For each of the above groups of experiments, three repetitions were set. When the ratio of the values of the positive and negative groups reached its maximum, the corresponding reaction conditions were considered the optimal reaction conditions for this detection method.

### Determination of the cut-off value

The cut-off value for the indirect ELISA was calculated using 142 clinical sera tested negative with both IFA and commercial kits. The average value ($\overset{-}{X}$) and standard deviation (SD) of the OD_450nm_ were calculated by statistical analysis. The cut-off value was determined as ($\overset{-}{X}$ + 3 SD). When the absorbance value of the serum samples at 450 nm was greater than the cut-off value, the serum samples were determined as positive. If it were below ($\overset{-}{X}$+2 SD), the serum samples were determined to be negative. If it was between ($\overset{-}{X}$ + 2 SD) and ($\overset{-}{X}$ + 3 SD), it was determined as suspicious.

### Sensitivity and specificity determination

The sensitivity of the method was determined by diluting SVA-positive serum from 1:2 to 1:8,192 according to the optimized conditions, reading the OD_450nm_ value, and observing the change in this value with increasing serum dilution. To estimate the specificity of established indirect ELISA, positive serum samples of PRRSV, CSFV, PCV2, PRV, PAstV, ASFV, PEDV, and SVA were detected under optimized conditions.

### Repeatability assay

To evaluate the repeatability of the indirect ELISA, the intra-batch and inter-batch repeatability of established indirect ELISA was determined using four SVA-positive serum samples and four SVA-negative serum samples. For intra-batch repeatability, each serum sample was repeated three times on ELISA plates coated at the same time. For inter-batch repeatability, each serum sample was repeated three times on ELISA plates coated with different batches. Results are expressed as the coefficient of variation (CV), which is the ratio of SD to the mean OD_450nm_ value for each group of serum samples.

### Stability assay

To test the stability of the indirect ELISA, ELISA plates coated with 3A protein were sealed in a 4°C refrigerator, and positive serum sample, negative serum sample, and PBS OD_450nm_ values were tested every 10 days. In addition, three replicates were set for each group. Finally, the stability of the indirect ELISA was evaluated by CV values.

### Preliminary application of the indirect ELISA in clinical serum samples

Parallelly, the commercial kit and IFA were used to detect 173 clinical serum samples of pigs at different growth stages, and IFA results were used as the standard to determine whether the serum samples were SVA-positive or negative. Subsequently, the 173 clinical serum samples were detected by the indirect ELISA, and all serum samples with OD_450 nm_ greater than 0.476 were regarded as anti-3A protein positive. When using this detection method to test clinical serum samples, a test result of less than or equal to 0.405 is considered negative, while a result between 0.476 and 0.405 is judged as suspicious. Finally, the results of the indirect ELISA, IFA, and commercial kits were compared to determine the effect of the indirect ELISA for detecting clinical serum samples.

## RESULTS

### Optimization of the indirect ELISA procedure

**Fig 1:**
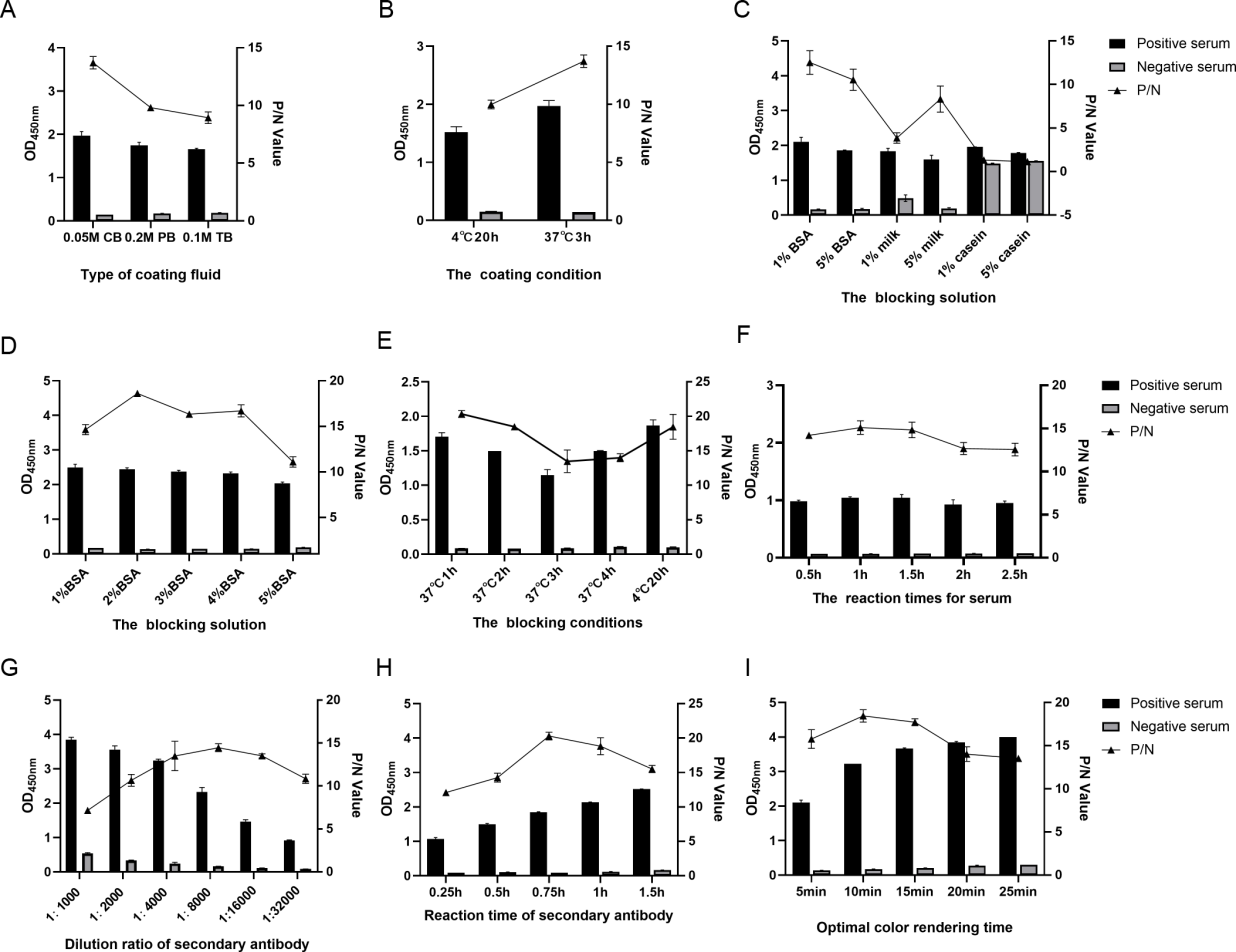
Optimization of the direct ELISA procedure. Determination of the (**A**) optimal type of coating fluid, (**B**) best coating condition, (**C**) best blocking solution, (**D**) optimal concentration of blocking solution, (**E**) optimal blocking conditions, (**F**) optimal incubation time for serum samples, (**G**) optimal dilution ratio of secondary antibody, (**H**) optimal reaction time of secondary antibody, and (**I**) optimal color rendering time. Data are presented as mean ± SD.

The optimal 3A protein coating concentration and the optimal dilution multiple of the serum sample to be tested were determined by chessboard titration method. The results showed that when the dilution of antigen and serum sample was 2 µg/mL and 1:100 in 100 µL per well, respectively, the ratio of positive (P) to negative (N) serum sample was the largest (P/N value was 17.971) (Table 1). Therefore, the final concentration of coated antigen is 200 ng/well, and the optimal dilution of serum sample is 1:100. The optimal conditions for the indirect ELISA were then determined by changing the conditions of each step of the ELISA operation. The results showed that the optimal conditions for the indirect ELISA were to dilute 3A protein with carbonate buffer (0.05 mol /L, pH 9.6) to 2 μg/mL and coat it for 3 h at 37°C, followed by 2% BSA solution for 1 h at 37°C (Fig. 1A through E). The serum samples were subsequently diluted at a ratio of 1:100 using PBS. The optimal incubation conditions for serum samples are to incubate at 37°C for 1 h (Fig. 1F). The optimal dilution ratio of the enzyme-labeled secondary antibody is 1:8,000, and the optimal incubation condition is to incubate at 37°C for 45 min (Fig. 1G and H). The optimal color development conditions were 10 min at room temperature and protected from light (Fig. 1I). During the optimization process of the indirect ELISA, the same set of positive and negative serum samples that had been tested by IFA and commercial serological test kits was used.

**TABLE 1:** Determination of optimal antigen coating concentration and serum dilutions^*b*^

Serum dilution ratio		Antigen coating concentration (µg/mL)				
		4	2	1	0.5	0.25
1:50	P	1.531	1.481	1.484	1.454	1.435
	N	0.214	0.213	0.212	0.211	0.210
	P/N	14.332	14.113	14.003	13.814	13.669
1:100	P	1.554	1.505	1.476	1.489	1.397
	N	0.181	0.168	0.183	0.172	0.191
	P/N	17.175	17.971^*a*^	17.192	16.306	14.657
1:200	P	1.305	1.285	1.265	1.247	1.225
	N	0.170	0.165	0.163	0.161	0.162
	P/N	15.300	15.612	15.554	15.523	15.124
1:400	P	1.145	1.104	1.082	1.041	1.022
	N	0.173	0.170	0.163	0.162	0.162
	P/N	13.268	12.971	13.309	12.846	12.631

^*a*^ P/N values of optimal serum sample dilution and optimal antigen-coated concentration.

^*b*^ P: OD_450nm_ value of positive samples. N: OD_450nm_ value of negative samples.

### Determination of cut-off value

**Fig 2:**
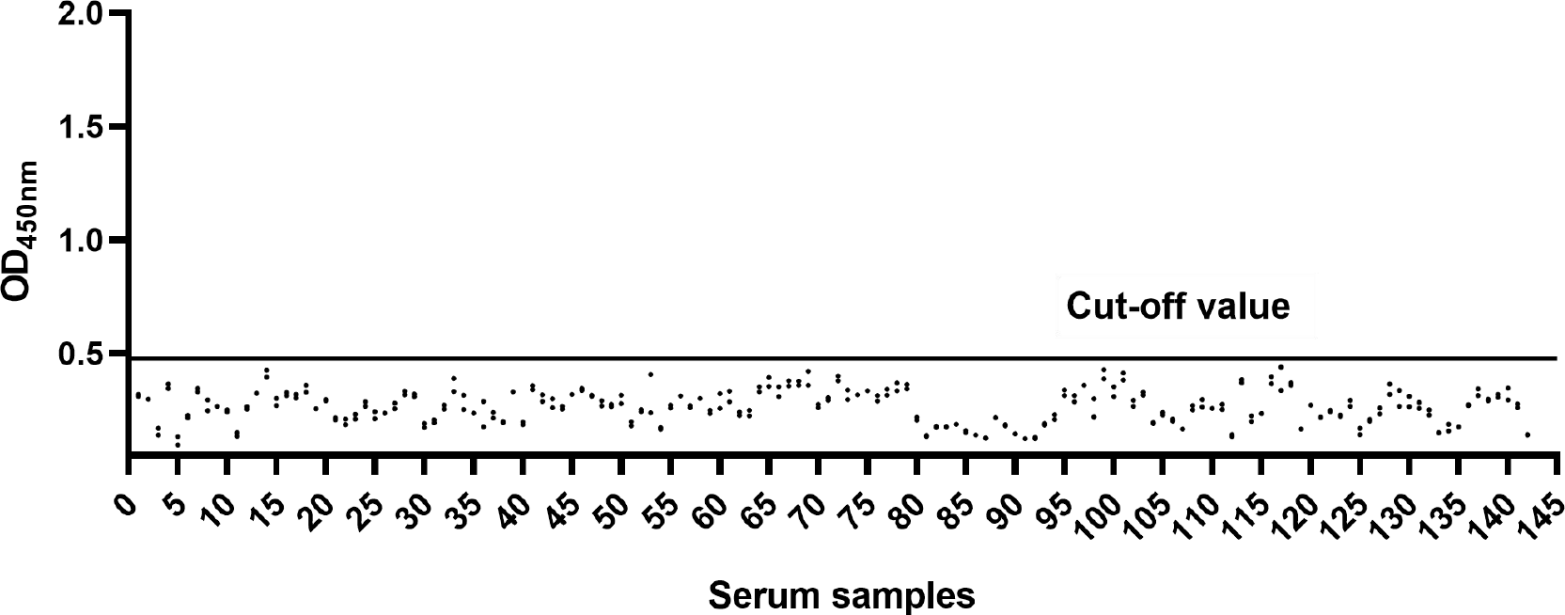
Using 142 serum samples that tested negative with both IFA and commercial kits, the cut-off value for the 3A indirect ELISA was determined (cut-off value = 0.476).

The optimized indirect ELISA was used to detect 142 porcine serum samples that had been tested negative by IFA and commercial kits. The results show that the average OD_450nm_ value was 0.263, and the standard deviation was 0.071. Finally, the cut-off value was calculated as $\overset{-}{X}$ + 3SD = 0.476 (Fig. 2). When the OD_450nm_ of the serum samples to be tested is greater than 0.476, it is considered as positive. The OD_450nm_ value of the serum samples was negative when it was less than $\overset{-}{X}$ + 2 SD (0.405). When the OD_450nm_ value of the serum samples is between 0.405 and 0.476, the serum samples are judged as suspicious. Re-testing is required if the test result is judged to be suspicious.

### Sensitivity and specificity assays of the indirect ELISA

**Fig 3:**
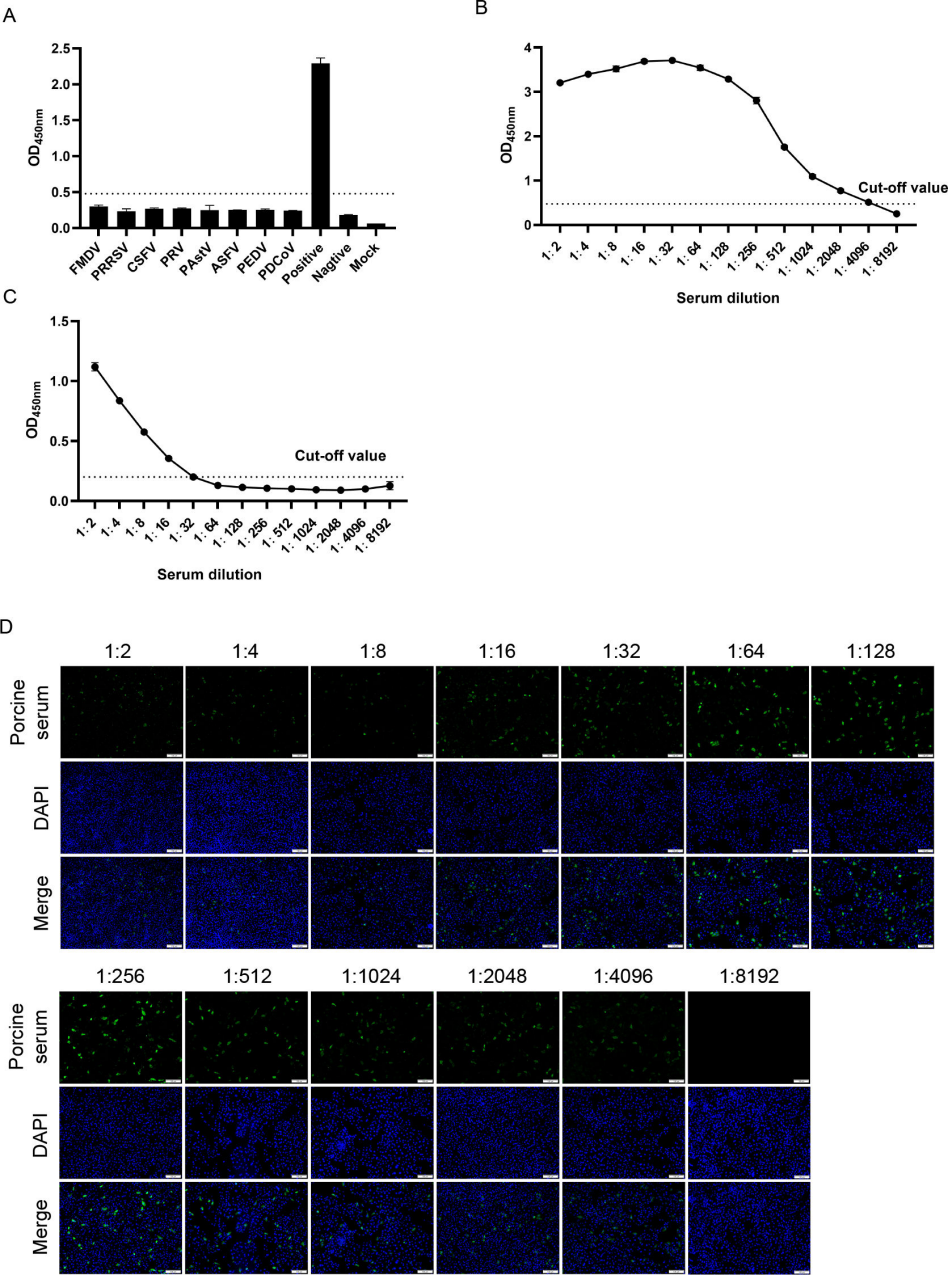
Performance evaluation sensitivity and specificity of the indirect ELISA. (**A**) Specificity test of the indirect ELISA. Positive: SVA-positive serum. Negative: SVA-negative serum. Mock: PBS. The indirect ELISA detected no cross-reactions with serum samples containing antibodies against eight other porcine pathogens, including FMDV, PRRSV, CSFV, PRV, PAstV, ASFV, PEDV, and PDCoV. (**B**) Determination of the indirect ELISA sensitivity. (**C**) Determination of commercial kits sensitivity. (**D**) Determination of IFA sensitivity. Data are presented as mean ± SD.

To evaluate the sensitivity of the indirect ELISA, the same SVA-positive serum sample was diluted to 1:8,192 for the assay. The result showed that the sensitivity of the indirect ELISA was 1:4,096 (Fig. 3B). Meanwhile, the sensitivity of the commercial kit was found to be 1:32, and that of IFA was 1:4,096 (Fig. 3C and D). Through comparison, the indirect ELISA has good sensitivity. Subsequently, to evaluate the specificity, the indirect ELISA method was used to detect PRRSV, CSFV, PCV2, PRV, PAstV, ASFV, PEDV-positive serum samples, as well as SVA-positive and -negative serum samples. The results showed that the OD_450nm_ values of all serum samples and SVA-negative serum were lower than the cut-off value, but the OD_450nm_ values of SVA-positive serum were higher than the cut-off value, indicating that the method has good specificity (Fig. 3A).

### Repeatability and stability assays

To evaluate the reproducibility of the indirect ELISA, we detected four SVA-positive serum samples and four SVA-negative serum samples by intra-batch and inter-batch reproducibility assays. The CV values obtained in both intra-batch and inter-batch reproducibility assays are below 10%, which indicates that the indirect ELISA has high reproducibility (Table 2). In addition, to verify the stability of the indirect ELISA, the kit was stored at 4°C and tested every 10 days. The CV values of SVA-positive serum group, SVA-negative serum group, and blank group were 10.69%, 14.4%, and 8.14%, respectively, which were all less than 15%. This result shows that the kit is stable and can be stored for at least 90 days at 4°C (Table 3).

**TABLE 2:** Results of the repeatability assay for the indirect ELISA^,^^*a*^

Sample no.		Intra-assay CV (%)		Inter-assay CV (%)	
		SD/$\overset{-}{X}$	CV (%)	SD/$\overset{-}{X}$	CV (%)
Positive samples	1	0.413/1.372	3.012%	0.035/1.373	2.556%
	2	0.088/3.062	2.883%	0.110/3.091	3.555%
	3	0.944/2.733	3.455%	0.055/2.728	2.013%
	4	0.019/1.585	1.238%	0.064/1.651	3.904%
Negative samples	5	0.003/0.160	1.848%	0.006/0.165	3.392%
	6	0.004/0.239	1.662%	0.003/0.244	1.212%
	7	0.010/0.230	4.289%	0.009/0.215	4.258%
	8	0.003/0.217	1.154%	0.004/0.171	2.635%

^*a*^ CV (%) = (SD / $\bar{X}$ ) × 100%.

**TABLE 3:** The indirect ELISA stability assay results

Day	Positive^*a*^	Negative^*b*^	Black^*c*^
10	3.338	0.266	0.081
20	3.222	0.255	0.085
30	2.829	0.250	0.076
40	2.760	0.281	0.080
50	2.341	0.186	0.073
60	2.580	0.237	0.072
70	3.012	0.332	0.076
80	2.646	0.257	0.075
90	2.646	0.235	0.062

^*a*^ Positive: OD_450nm_ value of positive sample.

^*b*^ Negative: OD_450nm_ value of negative sample.

^*c*^ Black: OD_450nm_ value of PBS.

### Detection of clinical serum samples

**Fig 4:**
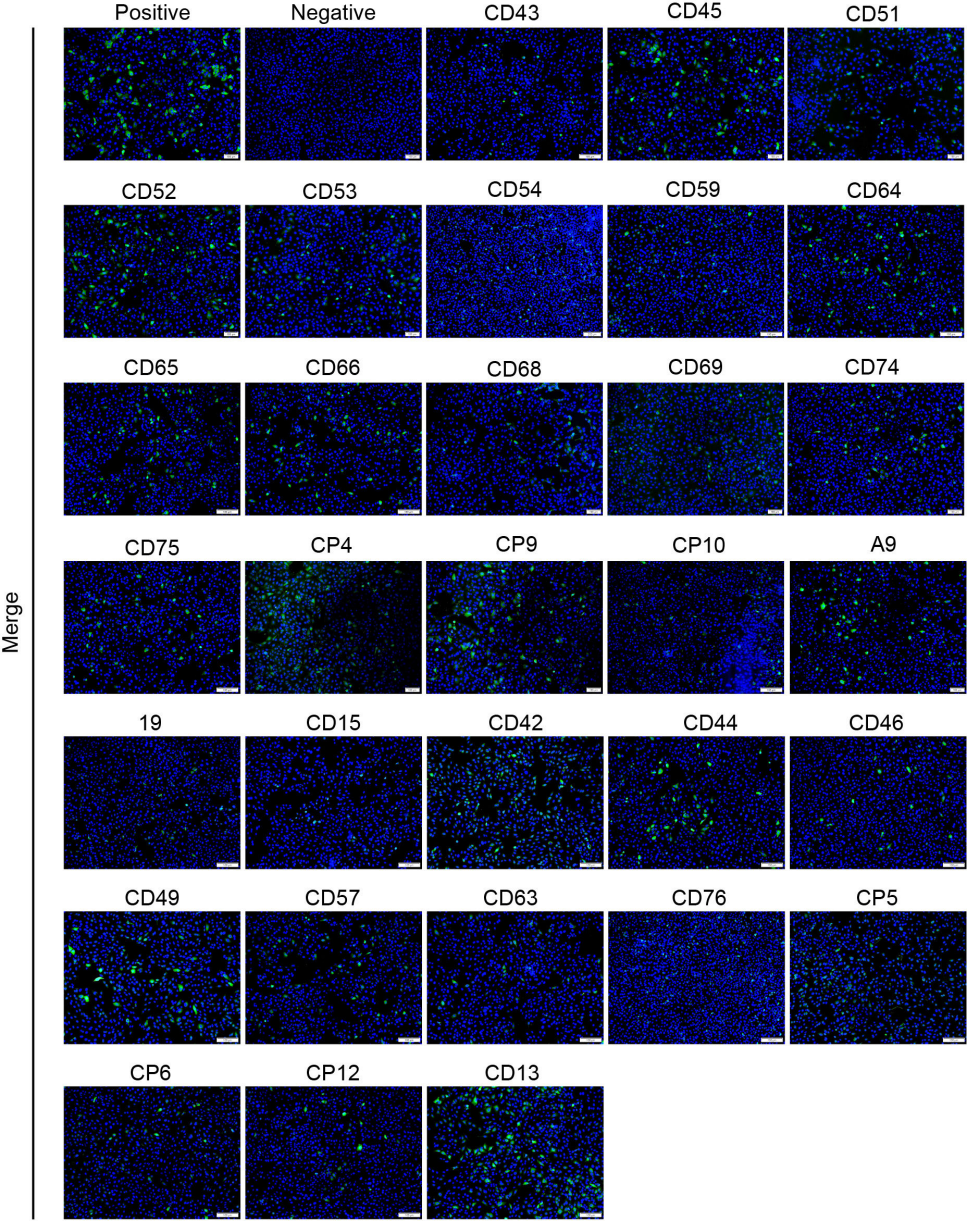
IFA detected 31 positive serum samples from 173 clinical serum samples. Positive: SVA-positive serum sample control. Negative: SVA-negative serum sample control. The 31 positive clinical serum samples were detected by 3A indirect ELISA and commercial kit, respectively. The results showed that serum samples 19, CD15, CD42, CD44, CD46, CD49, CD57, CD63, CD76, CP5, CP6, CP12, and CP13 were all positive by 3A indirect ELISA and commercial kit. Serum sample nos. CD43, CD45, CD51, CD52, CD53, CD54, CD59, CD64, CD65, CD66, CD68, CD69, CD74, CD75, CP4, CP9, CP10, and A-9 were all positive by 3A indirect ELISA and negative by commercial kit.

In order to evaluate the practical clinical application effect of the indirect ELISA, we used a commercial kit and IFA assay to detect 173 clinical serum samples from different stages of pig herds (Table 4). IFA test results showed that 142 of the 173 serum samples were negative and 31 were positive (Fig. 4). The commercial kits tested 160 serum samples as negative and 13 serum samples as positive. Compared with IFA results, the sensitivity and specificity of commercial kits were 100% (16/16) and 88.750% (142/160), respectively (Table 5). Subsequently, the 173 clinical serum samples were tested by the indirect ELISA method, and the results showed that the indirect ELISA and IFA detection results were consistent. Compared with the IFA results, the consistency rate of the indirect ELISA was 100%, while that of the commercial kit was 89.595%. The consistency rate between the commercial kit and the indirect ELISA was also 89.595% (Tables 6 and 7). Therefore, when testing clinical serum samples, the indirect ELISA has a higher detection performance than the commercial kit.

**TABLE 4:** Information on 173 clinical serum samples tested

Swinery	Positive	Negative	Sample quantity
35 days old	0	35	35
50 days old	0	17	17
65 days old	0	17	17
70 days old	0	16	16
95 days old	0	26	26
140 days old	0	15	15
Breeding boar	2	13	15
Pregnant sow	29	3	34
Total	31	142	173

**TABLE 5:** Comparison of commercial kit with IFA

Detection methods		IFA		
		Positive	Negative	Total
Commercial kit	Positive	13	0	13
	Negative	18	142	160
	Total	31	142	173
Data analysis	Sensitivity: 13/13^*a*^ = 100%			
	Specificity: 142/160^*b*^ = 88.750%			
	Coincidence rate: (13 + 142) / 173^*c*^ = 89.595%			

^*a*^ Sensitivity: the ratio of positive (13) to total (13).

^*b*^ Specificity: the ratio of negative (142) to total (160).

^*c*^ Coincidence rate: the ratio of the sum of positive (13) plus negative (142) to total (173).

**TABLE 6:** Comparison of the indirect ELISA with IFA

Detection methods		IFA		
		Positive	Negative	Total
3A indirect ELISA	Positive	31	0	31
	Negative	0	142	142
	Total	31	142	173
Data analysis	Sensitivity: 31/31^*a*^ = 100%			
	Specificity: 142/142^*b*^ = 100%			
	Coincidence rate: (31 + 142) / 173^*c*^ = 100%			

^*a*^ Sensitivity: the ratio of positive (31) to total (31).

^*b*^ Specificity: the ratio of negative (142) to total (142).

^*c*^ Coincidence rate: the ratio of the sum of positive (31) plus negative (142) to total (173).

**TABLE 7:** Comparison of the 3A indirect ELISA with commercial kit

Detection methods		Commercial kit		
		Positive	Negative	Total
3A indirect ELISA	Positive	13	18	31
	Negative	0	142	142
	Total	13	160	173
Data analysis	Sensitivity: 13/31^*a*^ = 41.94%			
	Specificity: 142/160^*b*^ = 88.750%			
	Coincidence rate: (13 + 142) / 173^*c*^ = 89.595%			

^*a*^ Sensitivity: the ratio of positive (13) to total (31).

^*b*^ Specificity: the ratio of negative (142) to total (160).

^*c*^ Coincidence rate: the ratio of the sum of positive (13) plus negative (142) to total (173).

## DISCUSSION

Since its discovery in 2002, SVA has spread rapidly in several countries, causing serious economic losses to the global pig industry (1, 2). Meanwhile, the clinical symptoms caused by SVA infection are very similar to those caused by FMD, SVD, and VS, making the prevention and control of SVA more difficult (1, 3).

Given that there is no commercially available vaccine for SVA in China, there is an urgent need for effective diagnostic tools to prevent and control its spread in China (6). As one of the main serological testing methods, ELISA exhibits high sensitivity, simplicity in operation, convenience, and rapidity, making it particularly suitable for the detection of clinical specimens (27). 3AB showed significant antigenic advantage in SVA-infected experimental animals (5). The 3A protein not only exists in an independent form during SVA infection but also functions as a component of 3AB protein, and considering that 3B protein has only 2.4 kDa, we hypothesized that 3A protein has good reactivity between SVA-positive serum samples (5, 40).

Meanwhile, the prokaryotic expression system is more suitable for large-scale production because of its higher protein expression efficiency, lower cost, and simpler operation than the eukaryotic expression system (42). Therefore, we established an indirect ELISA based on SVA 3A nonstructural protein. It was found that the sensitivity of the 3A indirect ELISA reached 1:4,096, which was better than that of the commercial kit (1:32). One hundred seventy-three porcine clinical sera were detected simultaneously by the 3A indirect ELISA, commercial kit, and IFA. When compared with the IFA test results, it was found that the concordance rate of the 3A indirect ELISA (100%) was higher than that of the commercial kit (88.75%) (22, 25). Therefore, the 3A indirect ELISA established in this study is a reliable and effective serological testing method for SVA, which can be used to enhance the surveillance and prevention of SVA epidemics in the long term.
